# Investigation of June 2020 giant Saharan dust storm using remote sensing observations and model reanalysis

**DOI:** 10.1038/s41598-022-10017-1

**Published:** 2022-04-12

**Authors:** A. Asutosh, V. Vinoj, Nuncio Murukesh, Ramakrishna Ramisetty, Nishant Mittal

**Affiliations:** 1 School of Earth, Ocean and Climate Science, Indian Institute of Technology Bhubaneswar, Bhubaneswar, Odisha 752050 India; 2 National Centre for Polar and Ocean Research (NCPOR), Ministry of Earth Sciences, Goa, 403804 India; 3 TSI Instruments India Private Limited, Bangalore, 560102 India

**Keywords:** Atmospheric science, Natural hazards

## Abstract

This paper investigates the characteristics and impact of a major Saharan dust storm during June 14th–19th 2020 on atmospheric radiative and thermodynamics properties over the Atlantic Ocean. The event witnessed the highest ever aerosol optical depth for June since 2002. The satellites and high-resolution model reanalysis products well captured the origin and spread of the dust storm. The Cloud-Aerosol Lidar and Infrared Pathfinder Satellite Observation (CALIPSO) measured total attenuated backscatter and aerosol subtype profiles, lower angstrom exponent values (~ 0.12) from Modern-Era Retrospective Analysis for Research and Application—version 2 (MERRA-2) and higher aerosol index value from Ozone monitoring instrument (> 4) tracked the presence of elevated dust. It was found that the dust AOD was as much as 250–300% higher than their climatology resulting in an atmospheric radiative forcing ~ 200% larger. As a result, elevated warming (8–16%) was observed, followed by a drop in relative humidity (2–4%) in the atmospheric column, as evidenced by both in-situ and satellite measurements. Quantifications such as these for extreme dust events provide significant insights that may help in understanding their climate effects, including improvements to dust simulations using chemistry-climate models.

## Introduction

Dust/Mineral dust is one of the important components of the atmospheric aerosols in the earth system. Dust contributes nearly 30% to the optical thickness and more than 70% to the total aerosol mass load^1^. The dust aerosol has both scattering and absorption characteristics in the solar and terrestrial radiation spectrum. It has the potential to perturb the radiation budget both by direct and indirect effects^2,3^. Dust possesses a broad range of impacts starting from local and global climate to human health^4–8^, biogeochemistry in the ocean^9,10^ and even on tropical cyclones^11^. Also, outbreaks of desert dust impact the air quality both locally and remotely^12,13^.

North Africa alone contributes more than 50% of global dust emission and is considered the active global dust source region^14,15^. African dust is known for its impacts on modulating West African rainfall^16^, providing nutrients for amazon rainforest^17^, health and public transportation^18^, as well as the development of Atlantic cyclogenesis^19,20^. During summer, the dust storm events are frequent over North-west Africa. The Saharan heat low (SHL) is the dominant atmospheric circulation pattern over North Africa^21,22^. The temperature gradient between the Gulf of Guinea (moist air) and inland SHL associated thermal winds develops the African Easterly Jet (AEJ). The AEJ usually peaks at 700 hPa and occurs north of 10 °N playing a vital role in the long-range transport of dust from storms over these regions^23^. Dust storm often occurs when a strong wind blows over loose sands. As a response, the dust gets injected high into the atmosphere. As dust reaches the level of AEJ^24^, it then gets transported to the west over the Atlantic Ocean^25,26^.

In June 2020, an anomalous pressure pattern developed over North Africa and the adjacent oceanic region due to the circumpolar northern hemispheric wave train^21^. This further intensified surface wind and AEJ. As a response, June 14–19, 2020, witnessed a massive dust storm over the Sahara Desert resulting in a huge amount of dust being lifted to the atmosphere. In particular, this storm was reported to be the strongest ever (June reference) since 2002. It reduced visibility across tropical Atlantic regions and got transported as far as the east coast of the USA, deteriorating the local air quality^27^. A recent study showed that the June 2020 historical dust storm facilitate an increase in SST and near-surface temperature over a sustained period over the study region^28^.

The existing ground-based measurements are not capable of monitoring the whole dust cycle, due to the large spatial distribution and the heterogeneous aerosol field over areas affected by dust plumes^29^. Hence satellite remote sensing could be an ideal method for studying the process of dust storms. Numerous studies have used satellite remote sensing datasets to investigate dust aerosols over regions like India^30,31^, China^32,33^, Africa/Sahara^21,34^, middle east^35^ and Australia^36^ like dust hotspots. In recent years, advancements in model reanalysis products add more information in detecting aerosol sources, their types and various impacts^5,37,39^.

Our study aims to characterise and investigate the impact of the giant Saharan dust storm during 14–19 June 2020. The state-of-the-art remote sensing datasets and model reanalysis are used to infer the possible changes in the aerosol properties, large scale radiative and thermodynamic effects. More details about the datasets used for this study can be found in the method section.

## Results and discussion

### Observation of the dust storm from space

**Figure 1:**
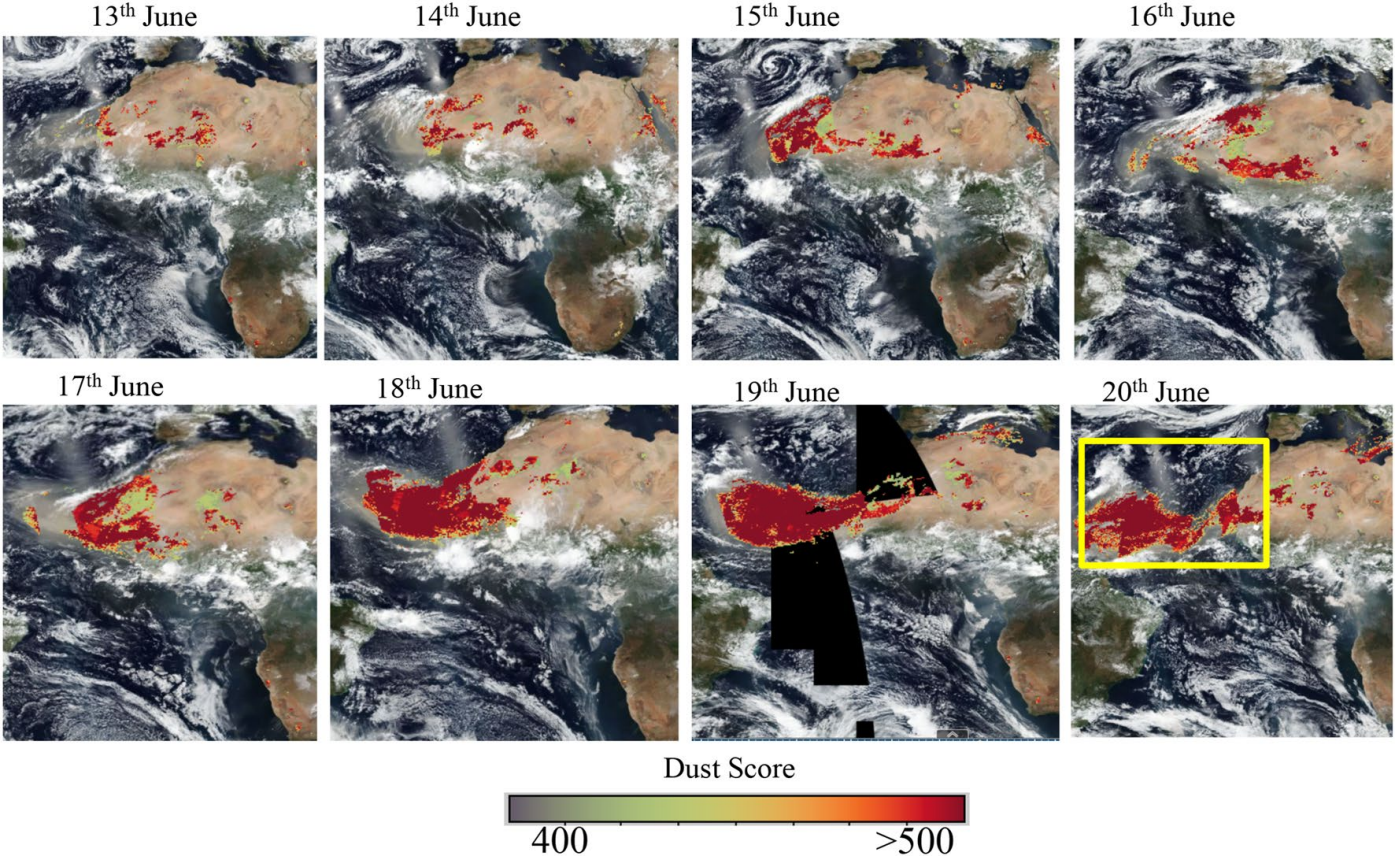
MODIS visible imagery indicating the evolution and transport of dust storm over the tropical Atlantic Ocean. The light brown colour indicates dust and the white shows clouds. The colour bar shows the value of the dust score from AIRS Level 2 datasets. The yellow box indicates the region of interest used for further analysis. These open-source datasets can be found in (https://earthdata.nasa.gov/). The map was generated using MATLAB 2015b, www.mathworks.com.

Figure 1 shows the time evolution of dust storm using the satellite image of Moderate Resolution Imaging Spectroradiometer (MODIS) and dust scores from Atmospheric infrared sounder (AIRS). The images are produced from the Earth Observing System Data and Information System (EOSDIS) worldview (https://worldview.earthdata.nasa.gov/). The dust storm started on 14th June and continued further until 19th June 2020. The high values of dust scores (> 400) show the spatial spread of the dust in the atmosphere. It may be observed that dust (brown colour) spread across a large region covering north-west African landmass and many parts of the tropical Atlantic Ocean covering several degrees of latitude and longitudes.

The Barcelona Supercomputing Centre-Dust Regional Atmospheric Modelling (BSC-Dream) simulations show higher values (ranges from 100 to 1000 μg m^−3^) of dust concentration over the north-western African regions (Supplementary Fig. S1). Such higher ranges are usually expected for severe dust storm cases as reported in earlier studies^6,7,40^.

### Multiplatform investigations of dust storm characteristics

**Figure 2:**
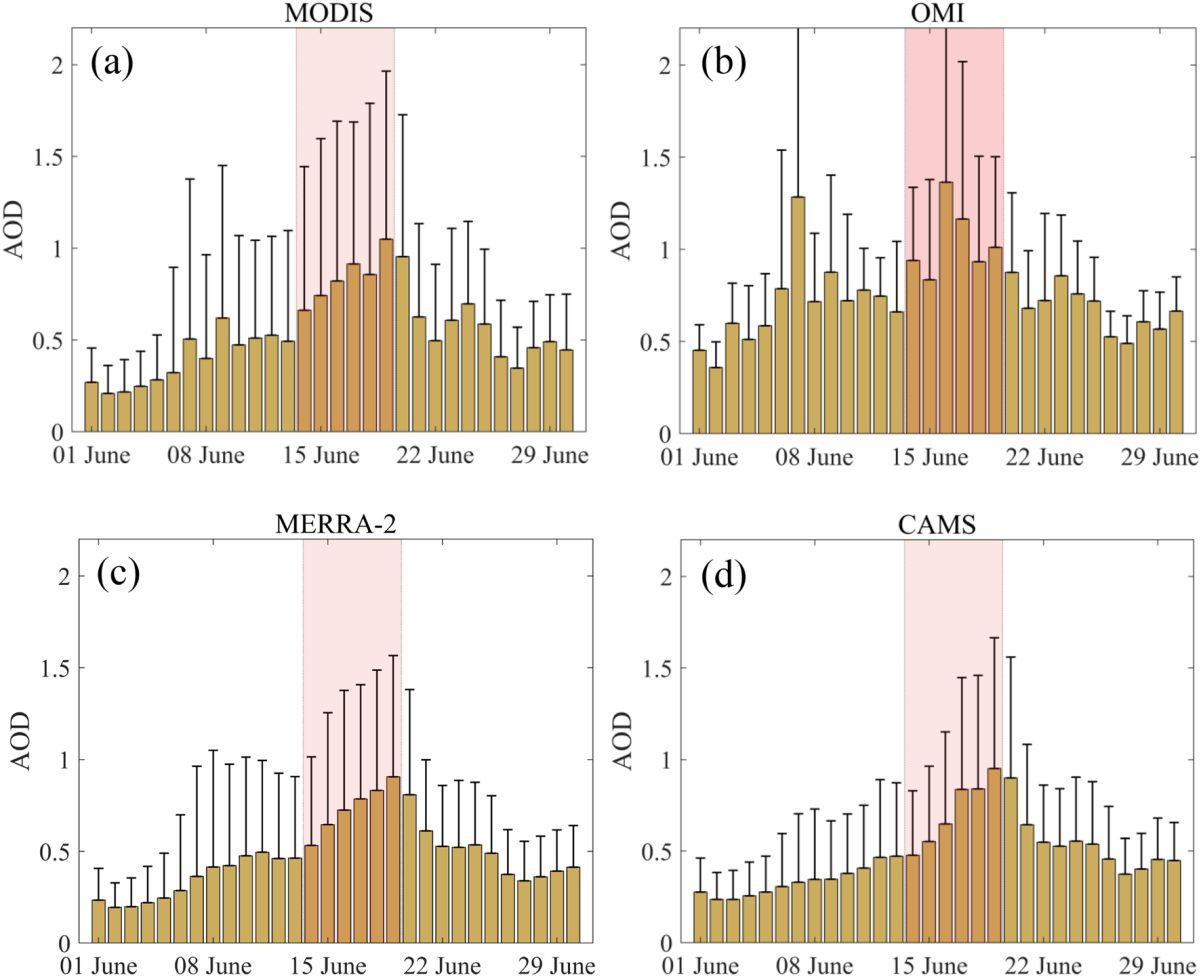
Area averaged time series of aerosol optical depth (AOD) during June 2020 from (**a**) MODIS, (**b**) OMI, (**c**) MERRA-2 and (**d**) CAMS. The shade is indicating the period of the dust storm (14–19, June 2020). The error bars indicate + 1σ of the daily datasets over the selected area of interest.

The Aerosol Optical Depth (AOD) is a measure of solar attenuation by the particles in the atmosphere (dust, smoke haze etc.). A higher value of AOD indicates higher loads of atmospheric pollutants. AOD has been used as a matrix to investigate dust storms and pollution studies^41–43^. The area-averaged (5 °N–30 °N, 50 °W–10 °W) AOD values for June 2020 is shown in Fig. 2 from four different platforms. The values in the shaded regions are representing the period of the dust storm. The high AOD values during 14–19 June 2020 support the start and intensification of the dust storm. The aerosol optical depth values from satellites (MODIS and OMI in Fig. 2a,b) show mean values higher than 1 and the maximum beyond 1.5. The OMI-AOD values are slightly higher than MODIS AOD possibly due to the spectral dependence of AOD as OMI (MODIS) measures AOD at 500 nm (550 nm). The higher AOD values indicate the severity of the storm. High values of AOD have also been reported during other dust storm events^31,43^. The reanalysis AOD (Fig. 2c,d) on the other hand, captures the dust event successfully; however, the values are a bit underestimated compared to the satellite observations. The maximum aerosol optical depth values are close to 1.5 for both MERRA-2 and CAMS reanalysis. The dust storm dissipated after 20th June 2020, with a drastic decline in AOD compared, to the storm period (Fig. 2).

### Inter-comparisons of AOD

**Figure 3:**
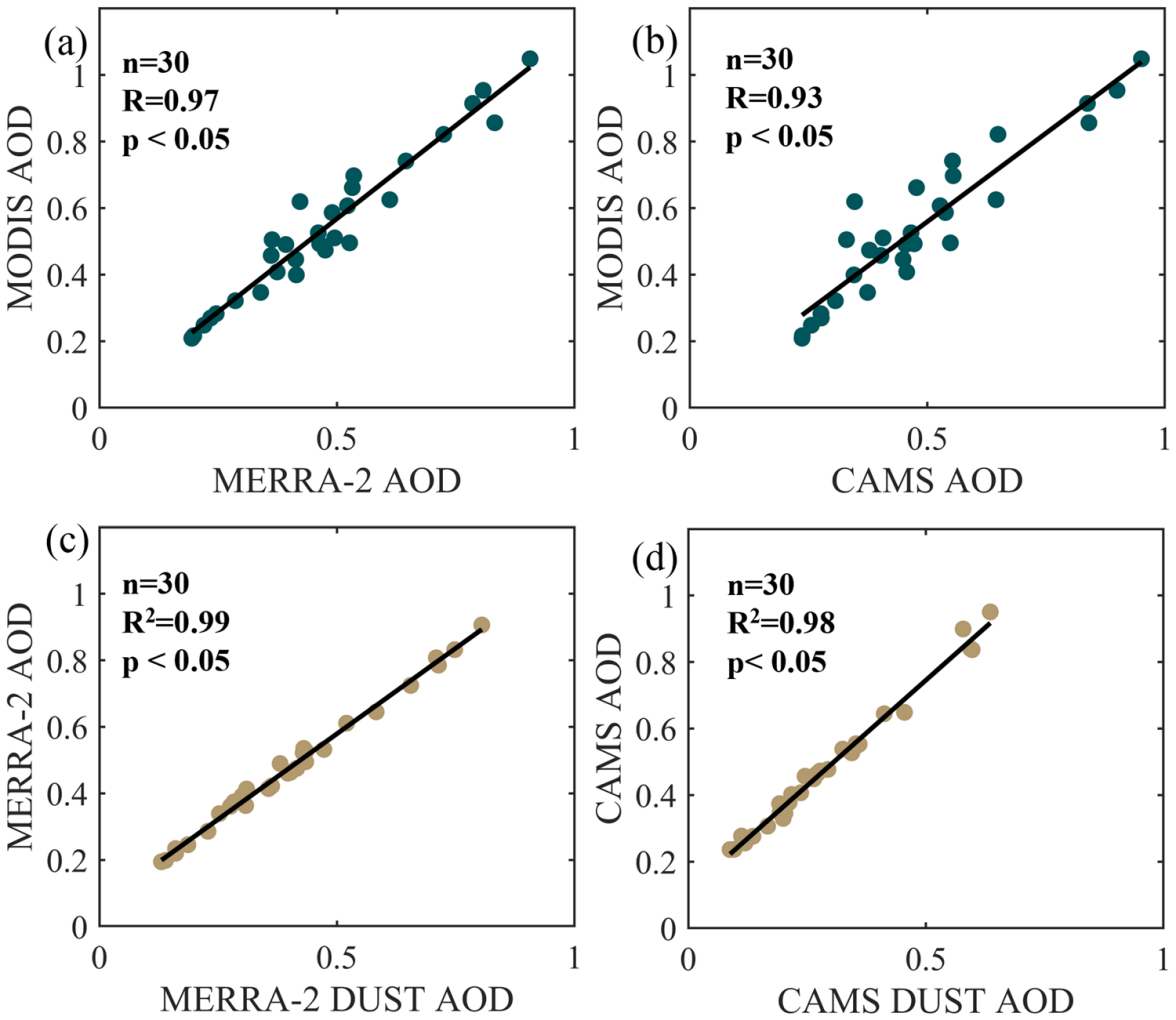
Correlations in AOD between (**a**) MODIS and MERRA-2, (**b**) MODIS and CAMS, (**c**) percentage variation in MERRA-2 AOD due to dust AOD (**d**) percentage variation in CAMS AOD due to dust. The datasets are used from 1st to 30th June 2020. The map was generated using MATLAB 2015b, www.mathworks.com.

The AOD from reanalysis (MERRA-2 and CAMS) are compared with MODIS AOD to investigate their variability during the study period (June 1–30, 2020) and are shown in Fig. 3a,b. It may be noted that both MERRA-2 and CAMS AOD show a statistically significant correlation with MODIS AOD (Pearson's r = 0.97 and 0.93 respectively). A high positive correlation indicates high confidence in the modelled/reanalysis AOD. This provides confidence that the reanalysis AOD also can serve better to investigate such high pollution episodes. The intensification of AEJ and surface winds could also have produced the sea salt AOD over the ocean. However, the regression analysis between reanalysis based total AOD and their corresponding DUST AOD (Fig. 3c,d) shows the dominance of dust over the study region (R^2^ = 0.99 and 0.98 respectively for MERRA-2 and CAMS reanalysis).

### ANG, AI and SSA characteristics

**Figure 4:**
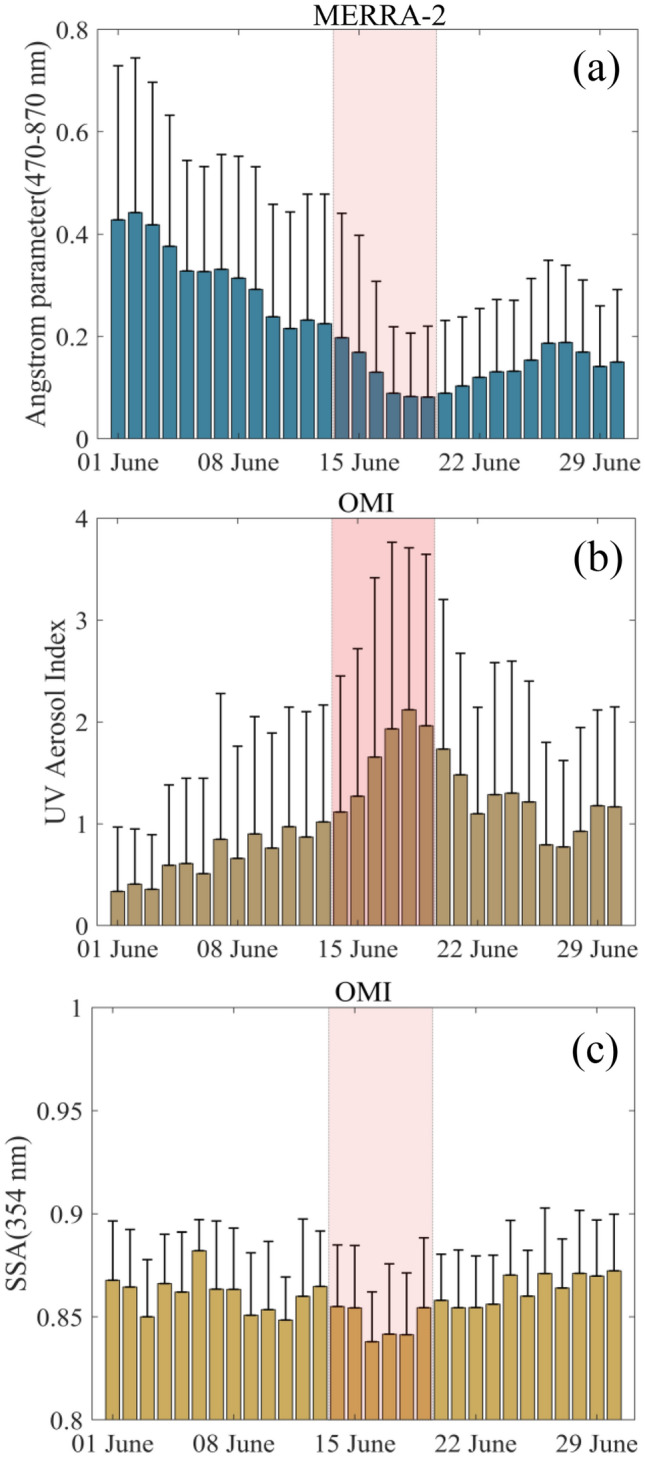
Area averaged time series of (**a**) Angstrom parameter from MERRA-2, (**b**) UV aerosol Index from OMI, (**c**) Single Scattering Albedo from OMI. The shading indicates the period of the dust storm. The error bars indicate + 1σ of the daily datasets over the selected area of interest. The map was generated using MATLAB 2015b, www.mathworks.com.

The angstrom parameter (ANG) explains the spectral dependence of aerosols. Additionally, it serves as a proxy for the size of the pollutant present in the atmosphere. Before the dust storm, the mean ANG was ~ 0.3 (Fig. 4a), which dropped to 0.12 during the dust storm event (Table 1). This indicates the dominance of coarser mode particles. The higher value of AOD (Fig. 2) and lower ANG values is a typical signature of dust (dominance of coarse mode) event as reported by many earlier studies^31,44,45^. Please note that the ANG (AOD) during this storm event was comparatively lower (higher) than the pre-dust storm values. This is obvious as atmospheric dust removals take a certain time and favourable conditions to happen. The prolonged drier atmosphere and stability (discussed separately) might have hindered the usual dust removal time. Also, this is normally a dust dominated region with a frequent incursion from the African deserts.

**Table 1:** Change in optical and thermodynamics parameters during dust storm w.r.to 2015–2019 climatology.

Satellite sensors	Model reanalysis	Variables	Event mean ± 1σ	Climatology (mean ± 1σ)	% Change (mean)
MODIS		AOD (550 nm)	0.91 ± 0.86	0.35 ± 0.23	160
OMI		AOD (500 nm)	1.15 ± 0.66	0.51 ± 0.23	125.5
	CAMS	AOD (550 nm)	0.71 ± 0.58	0.29 ± 0.16	144.8
		DUST AOD (550 nm)	0.53 ± 0.5	0.13 ± 0.12	307.7
	MERRA2	AOD (550 nm)	0.74 ± 0.63	0.27 ± 0.17	174.1
		DUST AOD (550 nm)	0.67 ± 0.6	0.19 ± 0.16	252.6
		ANG (470–800)	0.12 ± 0.18	0.3 ± 0.2	− 60
OMI		AI (360 nm)	1.68 ± 1.65	0.72 ± 0.61	133.3
OMI		SSA (354 nm)	0.84 ± 0.03	0.86 ± 0.27	− 2.3
	MERRA2	ARF TOA	− 20 ± 14	8.13 ± 5.22	153.4
		ARF BOA	− 36 ± 22	− 13.37 ± 7.6	169.3
		ARF ATM	15.4 ± 101	5.54 ± 3.65	193.9
AIRS		Temperature	21.7 ± 4	20.1 ± 3.18	8
AIRS		Relative humidity (RH)	64.5 ± 21.2	67 ± 20	− 3.7
Radiosonde		Temperature	18.39 ± 4.8	15.75 ± 2.77	16.8
		Relative Humidity (RH)	72.3 ± 15	73.9 ± 9.5	− 2.16

The aerosol Index (UV-AI or AI) along with AOD provides important information about absorbing aerosols (i.e., the smoke or dust) in the atmosphere. The AI values are averaged over the area same as AOD and presented in Fig. 4b. The AI values picked up just after 13th June and attained the maximum values in the ranges between ~ 2 to 4. Relatively higher AOD and AI (like this event) are signatures of a severe dust storm. Additionally, AI values of more than 3 indicate the presence of elevated dust^40,46,47^. The presence of high dust load during the dust storm is also being confirmed from our DUST AOD and column mass density analysis (Supplementary Figs. S2 and S3) and elevated dust observations using CALIPSO during the dust storm event (Supplementary Fig. S4).

The OMI single scattering albedo (SSA) values show wavelength dependence and also depend on the composition of aerosols^48^. It explains the nature of aerosol types (absorbing/scattering), present in the atmosphere. The OMI derived SSA is well verified with ground-based measurements for various environmental and dust storm conditions^49–52^. The single scattering albedo values (Fig. 4c) during the study period (except the storm event) were almost in the ranges > 0.86–0.9, which indicates the presence of absorbing background dust. During the storm period, the mean values of SSA dropped below 0.85, suggesting the addition of absorbing aerosols in the atmosphere. The SSA dropped almost 2% to its climatological mean values over the study region during the giant dust storm episode 2020 (Table 1).

### AOD during the dust storm

**Figure 5:**
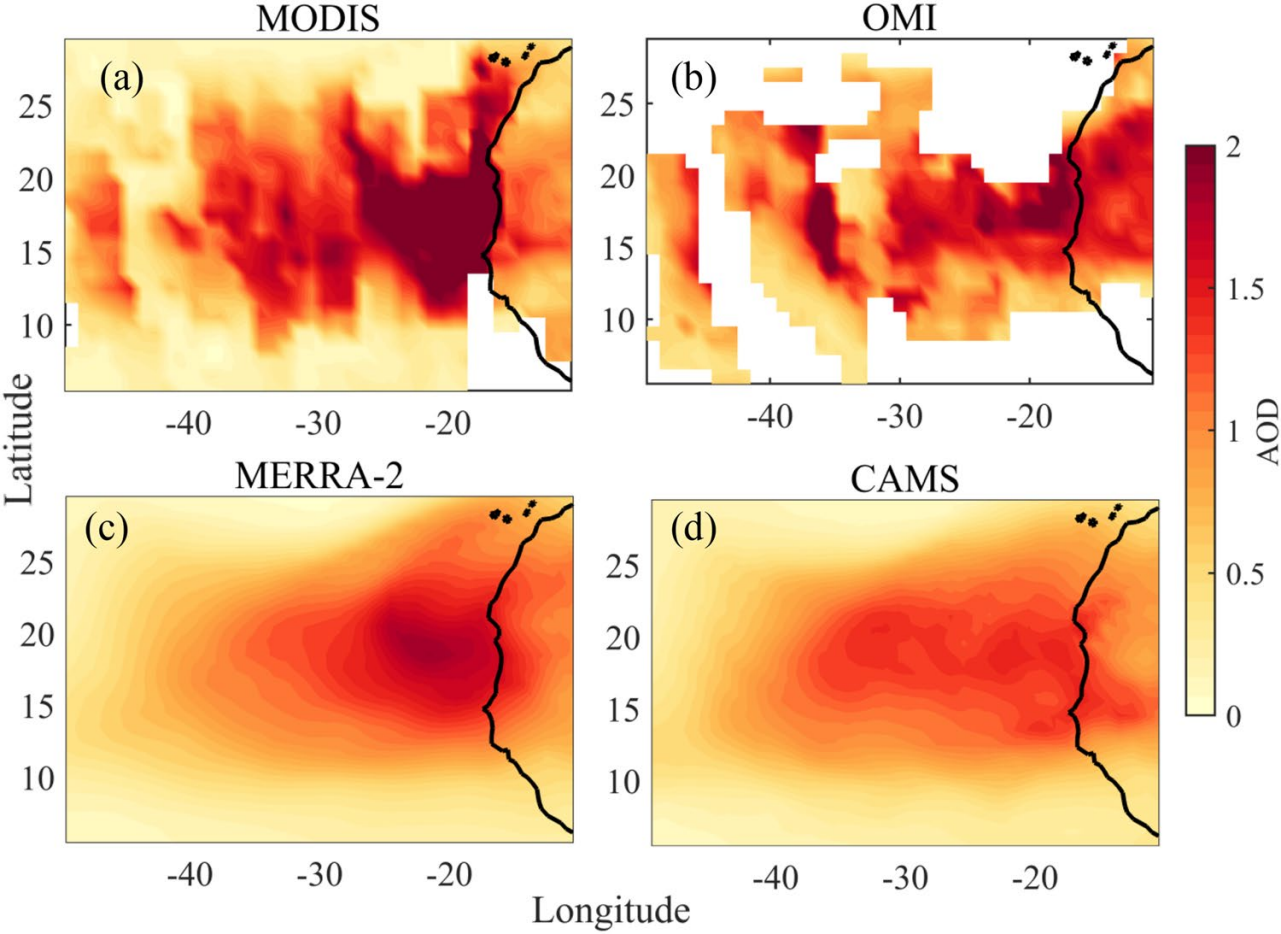
Time average spatial map of Aerosol optical depth (AOD) during the dust storm using datasets from (**a**) MODIS (**b**) OMI (**c**) MERRA-2 and (**d**) CAMS. The map was generated using MATLAB 2015b, www.mathworks.com.

The time-averaged cross-platform AOD values are shown in Fig. 5. The MODIS AOD (Fig. 5a) clearly shows the large longitudinal extent of the event. The mean AOD values for MODIS are > 2 close to coastal North-western Africa explains the high load of aerosol/dust and the severity of the storm. A similar signature is also visible in the OMI retrieved mean AOD (Fig. 5b). The model/reanalysis AOD (MERRA-2 and CAMS, Fig. 5c,d) also captures the spatial spread; however, the values are a bit underestimated as already discussed compared to the satellite observation which was also seen in area-averaged AOD values (Fig. 2).

### Aerosol radiative forcing

**Figure 6:**
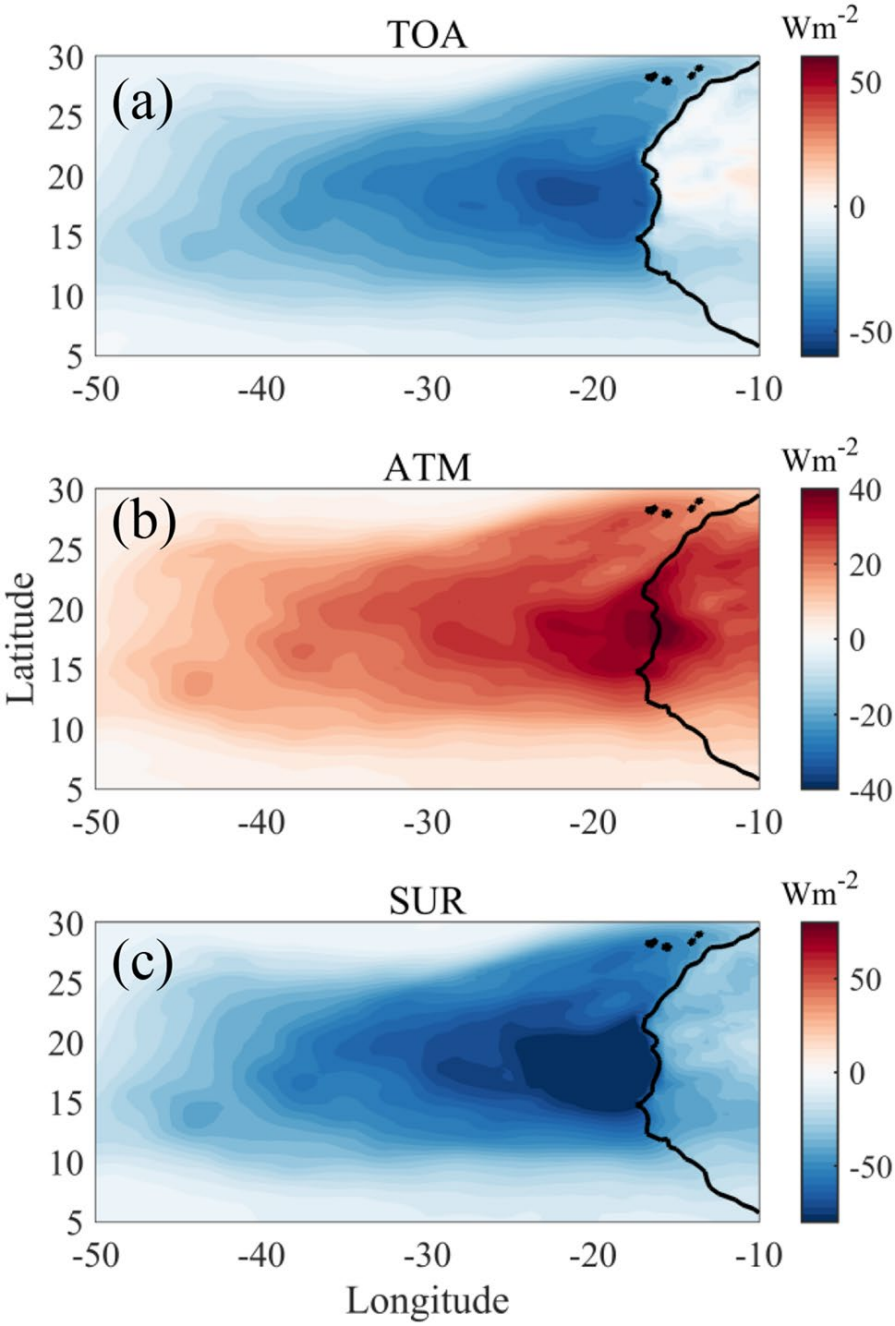
Spatial map (time-averaged) of ARF at (**a**) top of the atmosphere (**b**) in the atmosphere and (**c**) at the surface, using MERRA-2 datasets during the dust storm. The map was generated using MATLAB 2015b, www.mathworks.com.

The time-averaged (14–19 June 2020) aerosol radiative forcing (ARF) is estimated and shown in Fig. 6. The surface and top of the atmosphere, as well as surface ARF, are typically negative for dust over northwestern Africa and adjacent regions^53,54^. This particular storm witnessed maximum diurnal averaged ARF at the surface as high as – 150 W m^−2^_._ The values are found to be higher close to the coastal NW Africa and adjacent oceanic regions. Similarly, the TOA-ARF (Fig. 6a) has its maximum values ~ − 60 W m^−2^. In past, during the Saharan Dust Experiment (SHADE) measurement campaign, the peak radiative forcing was measured with a peak value up to – 130 W m^−2^^55^. A recent study^56^ based on the 2016 dust event in the Caribbean has reported shortwave radiative forcing of – 40 W m^−2^ at the surface and – 25 W m^−2^ at TOA. Our results are also comparable with the findings of Saidou Chaibou et al. 2020 over West Africa.

The high negative value at the surface and top of the atmosphere for this dust event suggests more attenuation of incoming solar radiation due to dust backscattering. The difference between the TOA and BOA ARF gives a positive forcing (absorbing) within the atmosphere with maximum ATM ARF that goes beyond 60 W m^−2^, suggesting a significant warming effect. Similar ranges (~ 70–100 W m^−2^) of positive atmospheric aerosol radiative forcing were reported before for other severe tropical dust storm events over India^57,58^.

ATM ARF for this dust event is ~ 200% higher (see Table 1) than its climatological mean, suggesting the dust intensification during the storm. Again, prolonged negative aerosol radiative forcing at the surface possibly may have an impact on the sea surface temperature over the ocean and the air-sea interactions. Also, the storm-induced change in the ATM ARF might have further affected the thermodynamics state of the atmosphere. We have discussed it separately in the subsequent section.

### Changes in the thermodynamics state variables in the atmosphere

**Figure 7:**
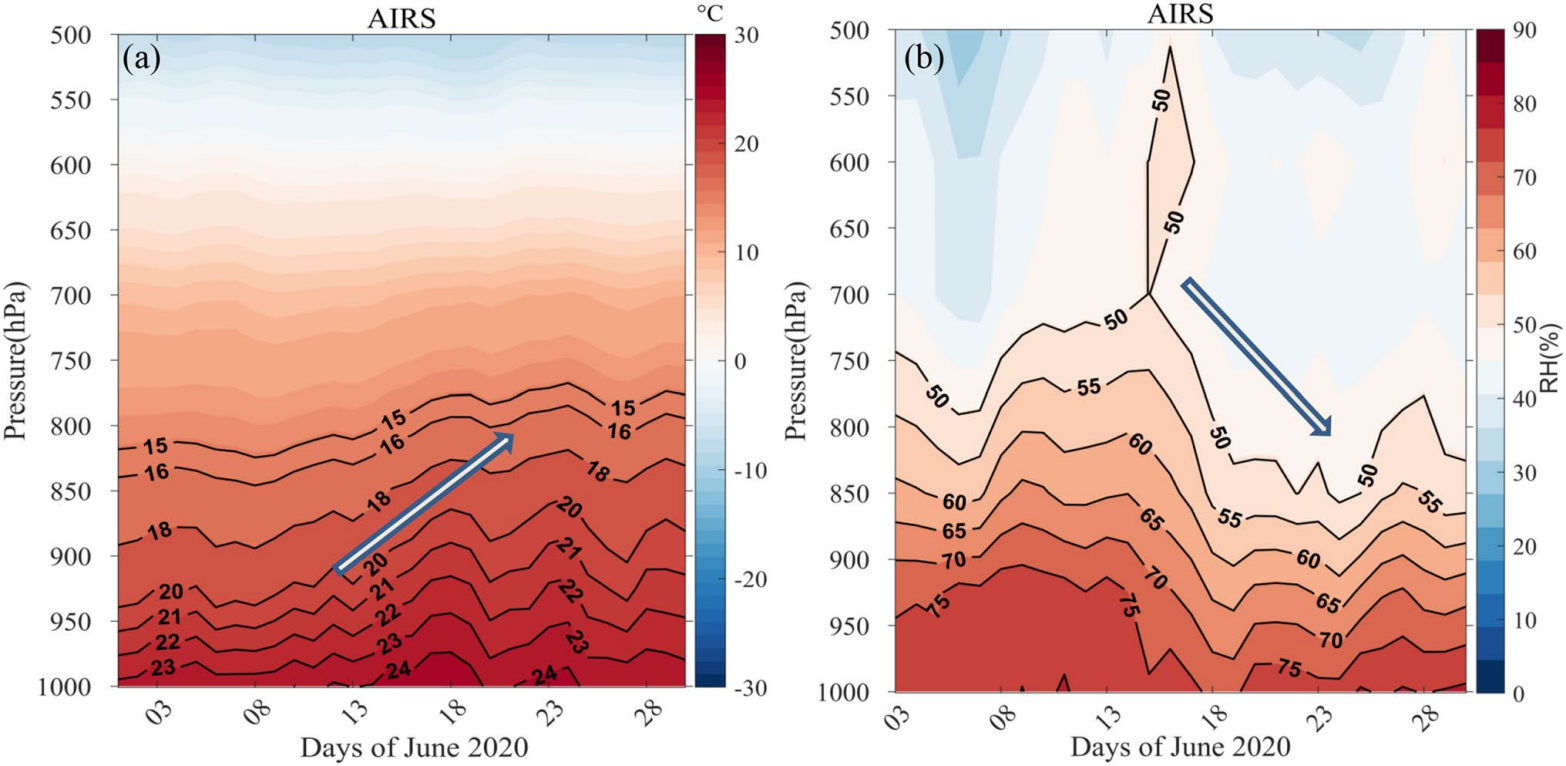
Area averaged time-height plot of (**a**) Temperature and (**b**) Relative humidity using AIRS during the dust storm. The map was generated using MATLAB 2015b, www.mathworks.com.

Temperature and relative humidity are two important thermodynamics variables of the atmosphere. Any change in the thermodynamic state can further impact the dynamics through changes to the thermal structure of the atmosphere. Here, we have examined the changes to these parameters using AIRS datasets (Fig. 7a,b). As mentioned earlier, an elevated dust layer that formed as a consequence of the dust storm, led to the warming of the atmospheric column due to the ARF response. The elevated warming signature is observed in Fig. 6a just coinciding with the dust storm (marked with an arrow). The warming effect is also observed from the surface to the mid-troposphere. The warming was found to be persistent even after the dust event, which could be due to the slow removal of dust from the atmosphere (Fig. 2 and Supplementary Fig. S2). At the same time, a sudden drop (please see the direction of the arrow) in the relative humidity was also observed collocated with the beginning of the storm event. This sudden drop in RH could be explained due to the increase in the dust induced atmospheric temperature/warming. The overall change in the mean temperature (averaged from surface to 850 hPa) is ~ 8% higher (the numbers are much higher if taken for specific dust levels) than its climatological values. A relative drop in RH (− 3.7%, Table 1) explains the significant impact of the dust storm. An attempt is also made to observe this warming using surface-based radiosonde measurements. The details are discussed in the next section.

### Regional changes

**Figure 8:**
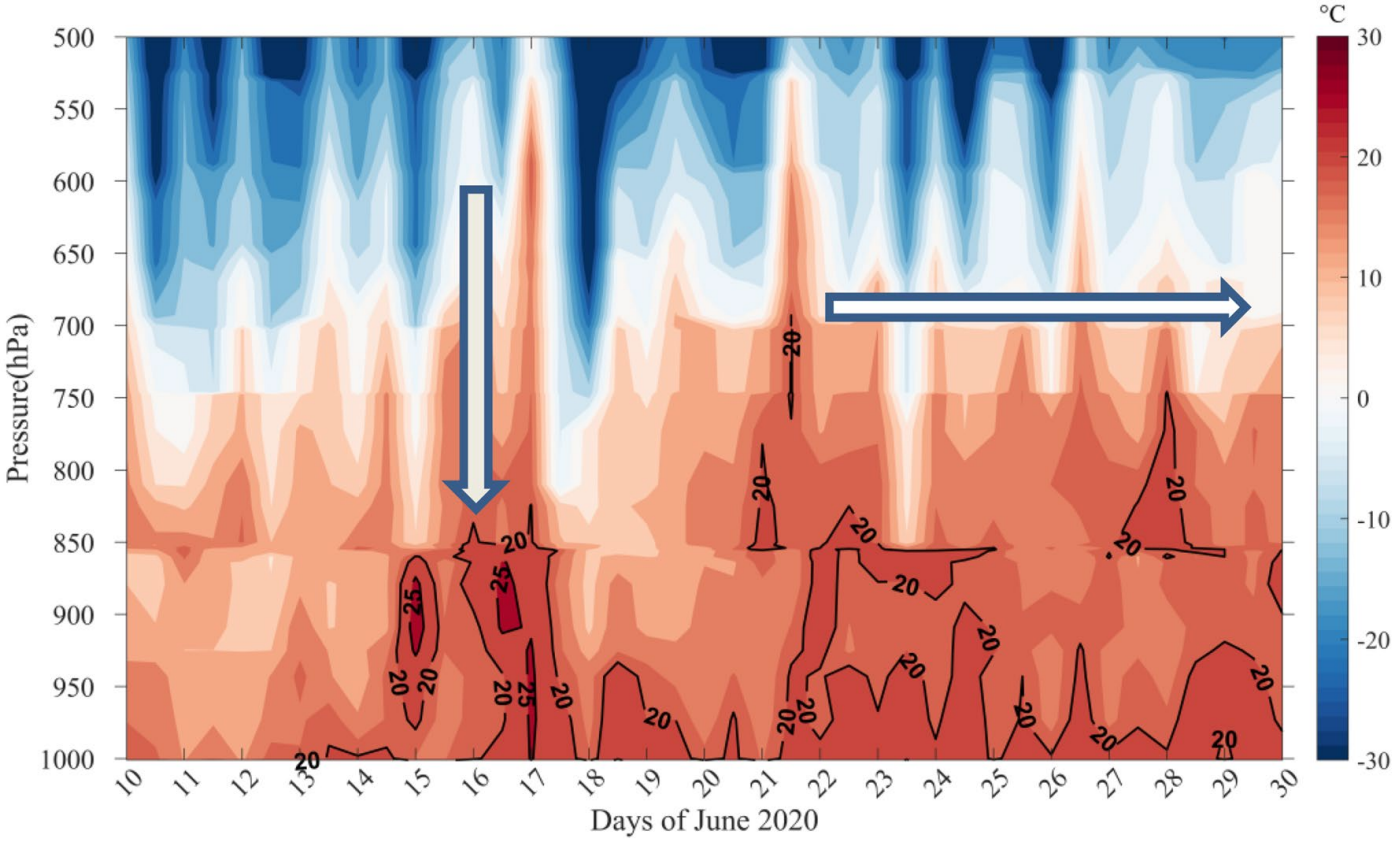
The time-height plot of temperature at Guimar-Tenerife (Station latitude: 28.47 °N Station longitudes: − 16.38 °W). The map was generated using MATLAB 2015b, www.mathworks.com.

The dust storm offers a unique opportunity to investigate the response of temperature and relative humidity regionally. We have used the sounding datasets at Guimar-Tenerife (Station latitude: 28.47 °N Station longitudes: − 16.38 °W) which falls within the study area. The time-height map of temperature (Fig. 8) shows a distinct signature of elevated warming. The lower atmospheric column was heated up with the beginning of the dust storm (14 June 2020) and the trend continued till the end of the month. The environmental conditions like higher surface winds^21^ and drier atmosphere helped the dust to remain in the atmosphere after the storm event which further amplified the post-storm warming as seen in Fig. 8. The elevated warming recorded in the radiosonde derived atmospheric profiles of temperature is similar to that observed in the AIRS measurements (Fig. 6a). The relative change in the temperature (averaged over the surface to 850 hPa) is ~ 16.8% higher than its climatological values (Table 1). It may be mentioned that atmospheric warming may be due to several reasons other than dust induced heating such as due to cloud formation, air mass incursion etc. The analysis clearly shows the sharp rise and fall in temperature coinciding with the dust storm. The reason for post-storm warming is hence not explored further. However, it is possible that the dust remained in the atmosphere for longer and also other atmospheric processes may have had a role in the post-dust storm warming.

**Figure 9:**
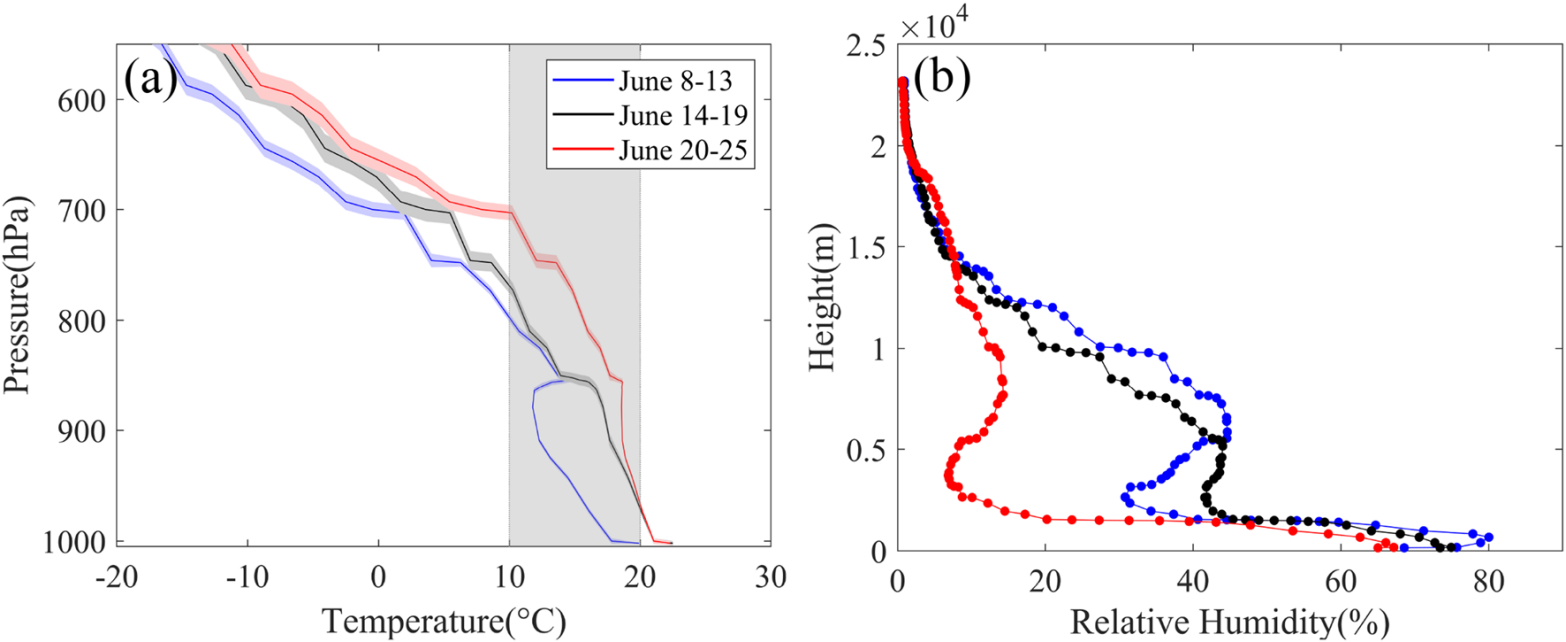
(**a**) Six days composite (day and night average) of pressure–temperature and (**b**) height-relative humidity profile (right) for pre, during the dust storm and post-event from radiosonde datasets for Guimar-Tenerife. The map was generated using MATLAB 2015b, www.mathworks.com.

We have further investigated the 6-day composite (before, during and after the dust storm event) of temperature and humidity profiles as depicted in Fig. 9. A clear distinction between pre and post-storm temperature profiles are visible. The temperature during the dust storm event (black line) is comparatively higher in the lower atmosphere than that of before storm composites. This indicates an elevated dust warming which might be due to the presence of a high dust load in the atmosphere. The post-storm composite (red line) is distinct in the higher altitude (700–900 hPa) compared to the event composite temperature whereas, the surface temperatures are close to each other. This signature is also clearly distinguishable in the time latitude temperature map (Fig. 8). The large decline in the whole column relative humidity points to the possibility of processes other than dust storm such as dry air incursion devoid of dust during this period.

The relative humidity profile in Fig. 9b also supports the dust induced warming signature at the lower levels of the atmosphere. Relative humidity drops with an increase in temperature. The post-event composite RH shows a remarkable drop and values are below 20% in the altitude of 0.5 to 1.5 km with maximum humidity change between the composites is observed at an elevation of 500 m from the surface.

Mostly, past studies for tropical dust storms (India and Saudi Arabia) reported enhancement in the near-surface humidity/relative humidity^59–61^ due to a decrease in surface temperature as a response to negative surface aerosol radiative forcing. On contrary, even there exists negative aerosol radiative forcing at the surface (Fig. 6), our study reviled that there was a net surface warming and a decrease in relative humidity associated with this dust storm over the study region (Figs. 7, 8 and 9). Using a numerical model simulation, Francis et al. 2022, found that along with SST, the air temperature rose about three times its climatological standard deviation (~ 1.8 K) due to this dust outbreak. Interestingly, from a 6-year (2012–2017) observational dataset, Milford et al.^12^ has also reported a drop in RH during dust outbreaks off the west coast of North Africa.

Such dust induced surface warming over the ocean and/or drop in relative humidity is contradicting with the findings of previous studies on the dust radiative impact and demand more scientific attention.

## Summary and conclusion

The present study is focused on characterising the radiative and thermodynamics impacts of the historical Saharan dust storm (by June standard) during 14–19 June 2020. Strong north-westerly near-surface winds triggered the event due to pressure distribution over part of the Atlantic Ocean and northwestern Africa. These features were reported to be part of a global circumpolar northern hemispheric wave train during June^21^. The dust storm event is investigated using state of the art satellite-derived products and high-resolution model reanalysis. The main findings of this work are summarized as follows.

1. The BSC-Dream model simulations, as well as satellite true colour images, showed the extreme nature of the dust storm that originated over the Sahara Desert.
2. The multiplatform analysis shows fair agreements between the model and satellite-derived AOD that showed values of as high as 2 during the event. More than 98% of variabilities in the total AOD were explained by Dust (Dust AOD) prove the presence of intense dust load in the atmospheric column. The spatial extent and magnitude between reanalysis and satellite AOD show good agreement with each other.
3. High values of AOD (~ 2) and low ANG (~ 0.1) were observed during the peak of the dust storm event (17 June 2020). The lower ANG values suggest the dominance of coarse mode dust particles in the atmosphere. The change in AOD was more than 150% (MODIS and MERRA2) whereas; DUST AOD change was ~ 250% (Table 1) compared to its climatology.
4. The higher value of UVAI observation signifies the presence of elevated dust. The CALIPSO data (Supplementary Fig. S4) also shows the vertical dust extent far up to 5 km altitude.
5. The maximum aerosol radiative forcing at the surface surged up to – 150 W m^−2^ and. and almost – 80 W m^−2^ at the top of the atmosphere near the dust source region. Such large radiative imbalances result from an atmospheric forcing/warming which is ~ 200% more than its mean climatology. Such a huge change in atmospheric radiative forcing is sufficient to affect atmospheric dynamics and thermodynamics.
6. The response of the dust storm is visible in the atmospheric thermodynamic state variables. There is more than a 16% increment in the temperature, and a 2% drop in relative humidity is observed (from the climatological mean) at a radiosonde site Guimar-Tenerife. A similar signature is also observed from AIRS satellite observation.

## Data and methods

We have used datasets from Moderate Resolution Imaging Spectroradiometer (MODIS)^62^, Atmospheric infrared sounder (AIRS)^63^, Ozone monitoring instrument^64^, Cloud-aerosol lidar and infrared pathfinder satellite observation (*CALIPSO*)^65^, Modern-Era Retrospective Analysis for Research and Application—version 2 (MERRA-2) reanalysis^66^, Copernicus Atmosphere Monitoring Service (CAMS) reanalysis^37^. Further, we have used the datasets of radiosonde provided by the University of Wyoming at the location Guimar-Tenerife^12^. All the datasets (except radiosonde data) are interpolated to MODIS resolution (1° × 1°) for comparison. All the analyses were carried out for the domain comprising north-western Africa and eastern to central Atlantic (5 °N–30 °N, 50 °W–10 °W, marked as a yellow box in Fig. 1). Brief details about the individual datasets are provided below.

### Moderate resolution imaging spectroradiometer (MODIS)

The Moderate Resolution Imaging Spectroradiometer (MODIS) as a part of Terra/Aqua satellites provides daily aerosol products worldwide^67,68^. With a view scan of ± 55°, it is present at orbit 700 km above the globe. It has spectral ranges of 0.41–15 μm at 36 different bands, ranging from visible to thermal IR^62,68^. The datasets are commonly used to study aerosol optical properties over both land and ocean surfaces. The daily mean of the Combined Dark Target and Deep Blue AOD at 0.55 μm for land and ocean (level 3) is used for this study. MODIS AOD is extensively used to investigate dust storms and other aerosol related studies^7,57,67^. More details about MODIS aerosol and other products can be obtained at http://modis.gsfc.nasa.gov.

### Ozone monitoring instrument (OMI)

The single scattering albedo (SSA), UV aerosol index (UVAI/AI, 354 nm) and AOD (500 nm) are used from the ozone monitoring Instruments (OMI). The instrument is on-board Aura satellite. It uses near UV (OMAERUV) algorithms for aerosol retrieval^64,69,70^. The original datasets have 0.25° × 0.25° spatial resolution and are level 3 global gridded products. The SSA plays an essential role in calculating aerosol radiative forcing. SSA ranges between 0 and 1 for entirely absorbing and completely scattering types of aerosols. The AI is calculated using spectral contrast at 331 and 360 radiance^69^. The AI is highly sensitive to absorbing aerosols and varies linearly with AOD^71^. OMI UV-AI provides important information towards investigations of aerosols as well as dust storm events^70,72^. The positive values of UVAI/AI indicate the presence of absorbing aerosols (like dust and smoke)^73,74^. On the other hand, negative values provide information about the dominance of scattering aerosols (e.g. sea salt, sulphate aerosols) in the atmospheric column^75^.

### Atmospheric infrared sounder (AIRS)

AIRS is a part of NASA's "A train satellite" and placed on Aqua satellite^63^. It provides accurate information about the atmospheric profiles of thermodynamics variables like temperature and humidity^76^. It also measures greenhouse gases like ozone, carbon dioxide, and methane. For this study, version 7, 1° × 1° resolution (latitude-longitude grids) datasets are used to investigate the relative humidity and temperature.

### Dust score

AIRS can be used to detect day and night dust properties using its longwave infrared channels dust-detection algorithm (DDA)^77^, These datasets can be used to calculate dust scores for detecting dust pixels over the ocean. Pixels, where the dust score is less than 360, are not shown in the figure. The numerical scale is a qualitative representation of the presence of dust in the atmosphere, an indication of where large dust storms may form. The sensor resolution is 45 km and the temporal resolution is daily.

### Cloud-aerosol lidar and infrared pathfinder satellite observation (CALIPSO)

The space lidar Cloud-aerosol lidar and infrared pathfinder satellite observation (CALIPSO) is widely utilized to study the vertical profile of dust and other aerosols worldwide^65,78^. The details about the retrieval algorithm can be found in Winker et al.^79^. CALIPSO has a 16 days repeat cycle and can observe aerosols over bright surfaces during clear and thin cloudy conditions. For this study, the CALIOP total attenuated backscatter (km^−1^ sr^−1^) and aerosol subtypes data products are used to investigate the vertical extent of dust during the storm event.

### MERRA-2 reanalysis

The Modern-Era Retrospective Analysis for Research and Application—version 2 (MERRA-2) provides data beginning in 1980^80^ following the original MERRA reanalysis. The Goddard Earth Observing System-5 (GEOS-5) atmospheric general circulation model with 3DVar data assimilation system is used to prepare MERRA-2 datasets^81^. The GEOS-5 model resolution is roughly 0.5° × 0.625° in latitude and longitude, with 72 hybrid-eta layers. The aerosol data assimilation uses reflectance from the Advanced Very-High-Resolution Radiometer (AVHRR) sensor (1979–2002)^82^, MODIS on Terra and Aqua, AOD retrievals from MISR (2000–2014)^83^ and aerosol measurements from AERONET^84^. We have used the AOD, Dust AOD, Angstrom parameter (ANG) and radiative forcing parameters from the MERRA-2 reanalysis.

### CAMS reanalysis

The CAMS (Copernicus Atmosphere Monitoring Service) datasets is the largest global reanalysis datasets for atmospheric compositions^85^. It uses the ECMWF's Integrated Forecasting System (IFS), with 60 hybrid sigma/pressure levels along with a 4DVAr data assimilation procedure. The IFS uses 12 prognostic variables (11 aerosol mass mixing ratios and one precursor—SO_2_) and assimilates both the satellite and in situ data. The model uses various schemes for simulating Dust, sea salt and other gaseous precursors. The assimilated observations include AOD from the MODIS instruments onboard the Terra and Aqua satellites, both over the ocean and dark land surface. The CAMS AOD is well validated by independent observations and with satellite datasets^37^. AOD and Dust AOD from CAMS are used in this study.

### Radiosonde observations

The radiosonde observation data (00Z and 12Z) provided by the University of Wyoming^12^ is used to study the upper atmospheric thermodynamic state (i.e., temperature and relative humidity structure). We utilize the observations from Guimar-Tenerife (Station latitude: 28.47 °N Station longitudes: − 16.38 °W) and is close to the dust storm's origin.

### DREAM model simulations

DREAM (Dust Regional Atmospheric Modelling) is a 3D model that simulates all major processes (emission, transport and removal) of mineral dust aerosol^86^. The Barcelona Supercomputing Centre (BSC) made the model simulations available, hence popularly called the BSC-DREAM model. The model uses the thermal state of the atmosphere, near-surface winds, soil properties, and vegetation covers etc., to simulate dust. The model has proven accuracy in predicting dust storm events^6,7,87–89^ and is well-validated with datasets from various satellite observations and observational networks^6,90,91^.

### Aerosol radiative forcing (ARF) calculation

The presence of aerosols over a region interacts with the radiative balance in various ways. ARF is the change in solar and terrestrial flux with and without the aerosols. The strength and nature of ARF at the top (TOA) bottom (SUR) and in the atmospheric column (ATM) have various environmental implications^38^. MERRA-2 simulated radiative fluxes have shown good agreements with CERES satellite radiation data products^92^. To calculate the clear sky aerosol radiative forcing using MERRA-2 fourth assimilation stream hourly data (MERRA-2_400.tavg1_2d_rad_Nx), we adopted the methodology from Penna et al.^38^. The mathematical representation of the calculation of ARF is as follows

1 $${ARF}_{SUR}=\left(\mathrm{SWGNTCLR}+\mathrm{LWGNTCLR}\right)-\left(\mathrm{SWGNTCLRCLN }+\mathrm{LWGNTCLRCLN}\right),$$

2 $${ARF}_{TOA}=\left(\mathrm{SWTNTCLR}+\mathrm{LWTUPCLR}\right)-\left(\mathrm{SWTNTCLRCLN }+\mathrm{LWTUPCLRCLN}\right),$$

3 $${ARF}_{ATM}= {ARF}_{TOA}-{ARF}_{SUR}.$$

SW/LW stands for shortwave/ longwave, GN/TN stands for surface and top of the atmosphere net radiation flux, whereas CLR/CLN stands for clear sky and clear sky with no aerosols respectively. More details can be found in Penna et al.^38^ and Sanap et al.^39^.

To calculate the climatology, a total of 5 years (2015–2019) of each dataset have been used covering the dates of the events.

## Supplementary Information

Supplementary Figures.

## Data Availability

Datasets are freely available and can be downloadable from the internet. The codes and datasets used in this study can be shared upon request to the corresponding author.
