# Supplementary material for: Investigation of June 2020 giant Saharan dust storm using remote sensing observations and model reanalysis
Source: Sci Rep. 2022 Apr 12;12:6114. doi: 10.1038/s41598-022-10017-1 (PMC9005708; doi:10.1038/s41598-022-10017-1)
Supplement: Supplementary file 1 — Supplementary Figures. [file 41598_2022_10017_MOESM1_ESM.docx]

**Investigation of June 2020 giant Saharan dust storm using remote sensing observations and model reanalysis**

A. Asutosh^1 *^, V. Vinoj^1^, Nuncio Murukesh^2^, Ramakrishna Ramisetty^3^ and Nishant Mittal^3^

^1^School of Earth, Ocean and Climate Science Indian Institute of Technology Bhubaneswar, Bhubaneswar, Odisha, India,752050

^2^National Centre for Polar and Ocean Research (NCPOR), Ministry of Earth Sciences, Goa, India,403804

^3^TSI Instruments India Private Limited, Bangalore, India, 560102

Correspondence: [asutosh.acharya@gmail.com](mailto:asutosh.acharya@gmail.com)

**Supplementary Figures**

Fig.S1: BSC-DREAM model simulated surface dust concentration (μg/m^3^) over the dust source region.

Fig S2: Area averaged time series for (a) Dust AOD from MERRA-2 (b) dust AOD from CAMS.

Fig S3: Area averaged Dust column Mass density from MERRA-2(left) and temporal average during the event period (right).

Fig S4: The total attenuated backscatter (km^−1^ sr^−1^) and aerosol types over the study region during the dust storm event from CALIPSO observation.
